# New hairworm (Nematomorpha, Gordiida) species described from the Arizona Madrean Sky Islands

**DOI:** 10.3897/zookeys.733.22798

**Published:** 2018-02-01

**Authors:** Rachel J. Swanteson-Franz, Destinie A. Marquez, Craig I. Goldstein, Andreas Schmidt-Rhaesa, Matthew G. Bolek, Ben Hanelt

**Affiliations:** 1 Center for Evolutionary and Theoretical Immunology, Department of Biology, 163 Castetter Hall, MSC032020, University of New Mexico, Albuquerque, New Mexico 87131-0001, USA; 2 Rush Oak Park Hospital, Department of Emergency Medicine, 520 South Maple Avenue, Oak Park, Illinois 60304, USA; 3 Zoological Museum and Institute, Biocenter Grindel, Martin-Luther-King-Platz 3, University of Hamburg, 20146 Hamburg, Germany; 4 Department of Integrative Biology, 501 Life Sciences West, Oklahoma State University, Stillwater, Oklahoma 74078, USA

**Keywords:** Nematomorpha, Gordiid, hairworm, *Gordius*, Sky Islands, new species

## Abstract

Gordiids, or freshwater hairworms, are members of the phylum Nematomorpha that use terrestrial definitive hosts (arthropods) and live as adults in rivers, lakes, or streams. The genus *Paragordius* consists of 18 species, one of which was described from the Nearctic in 1851. More than 150 years later, we are describing a second *Paragordius* species from a unique habitat within the Nearctic; the Madrean Sky Island complex. The Madrean Sky Islands are a series of isolated high mountains in northern Mexico and the southwestern United States (Arizona and New Mexico), and are well known for their high diversity and endemicity. The new species is described based on both molecular data (COI barcoding) and morphological characters of the eggs, larvae, cysts, and adults. Adult females have unique small oblong mounds present on the interior of the trifurcating lobes with randomly dispersed long hairs extending from the furrows between the mounds. Marked genetic differences support observed morphological differences. This species represents the second new hairworm to be described from the Madrean Sky Islands, and it may represent the first endemic hairworm from this biodiversity hotspot.

## Introduction

Hairworms are in the phylum Nematomorpha, belonging to one of only 3 entirely parasitic metazoan phyla (Hanelt et al. 2005). Nematomorphs are arthropod parasites with indirect lifecycles, infecting aquatic insect larvae as their paratenic hosts, and orthopterans, coleopterans, or mantids as their definitive hosts (Hanelt et al. 2005). Worms are free living in aquatic environments as adults, where mating and oviposition occur. Larvae subsequently are swallowed by and encyst in suitable paratenic hosts such as midge larva (Hanelt and Janovy 2004a). Terrestrial definitive hosts are infected upon the consumption of infected adult aquatic insects. Upon maturation, the hairworm will alter host behavior so that it becomes water seeking (Biron et al. 2005). Once the definitive host enters the water, the horsehair worms will emerge and begin mating, completing the lifecycle.

Hairworms have been chronically understudied. One estimate suggests that only 14% of species have so far been described (Poinar 2008), and most descriptions have been limited to the Palearctic. Within the Nearctic, one area requiring biodiversity work is the desert Southwest. For example, despite its diverse array of 13 biomes, supporting a wide range of biotic and abiotic habitats, only 3 species have been recorded from the southwestern state of Arizona: *Paragordius varius* (Leidy, 1851), *Gordionus violaceus* (Baird, 1853) and *Pseudochordodes gordioides* (Montgomery, 1898) as well as a yet to be described and named *Gordius* sp. from the Chiricahua mountains, part of the Sky Islands (Hanelt et al. 2015). We believe that this depauperate biodiversity is due to lack of study, and we have focused on investigating the northern tip of the Madrean Sky Island chain in southern Arizona.

## Methods

### Field collections

First field collections occurred on 27 July, 2011, at a stream in the Huachuca Mountains, Sunnyside, Cochise Country, Arizona, USA (31.445, -110.402, elevation: 1770 m). Subsequent collections were made on 28 July, 2011, from stream puddles near mile marker 12 on Madera Canyon Road, Madera Canyon, Santa Rita Mountains, Santa Cruz County, Arizona, USA (31.713, -110.87, elevation: 1640 m). All specimens were collected as free-living adults and transported alive, in stream water, to the laboratory. In the laboratory and before adult worms were processed for morphological and molecular analyses, worms were allowed to mate and females were allowed to deposit egg strings.

### Biological material and microscopy

**Adults**. Physical attributes of specimen length and color were recorded in the laboratory. Measurements were obtained by placing specimens on a metric ruler, taking precautions to not stretch specimen. Specimens were cut into four pieces using razor blades. Pieces from the anterior, posterior, and mid-section were preserved in 70 % ethanol at room temperature for future microscopy work. The remaining mid-section pieces were preserved in 100 % ethanol at -80 °C for future molecular analysis. Tissue samples preserved for microscopy were imaged using a Scanning Electron Microscope (SEM). Specimens were cleaned of debris using a previously-described method (Salas et al. 2011). Briefly, two drops of Clinique make up remover (Clinique, New York City, New York) placed into 1.5 ml tubes containing 70 % ethanol. The 1.5 ml tubes were placed into iSonic® Ultrasonic Cleaner Model D7810A (iSonic Inc., Chicago, Illinois), and cleaned for 4 minutes at maximum speed. Specimens were prepared by placing them in four increasing concentrations of ethanol (70 %, 85 %, 95 %, 100 %). Specimens were then dehydrated by placing them in increasing concentrations of hexamethyldisilazane (HMDS). Tissues were then mounted on stubs with carbon tape and coated with gold-palladium in an EmiTech K950 turbo-pumped vacuum coater with the gold-palladium sputter coater attachment (Quorum Technologies, West Sussex, England). Observations were made and digital images were taken using a JEOL 5800LV SEM at 15 kV (JEOL Ltd., Tokyo, Japan).

**Eggs and larvae using light microscopy.** For egg and larval measurements, pieces of egg string and hatched larvae were prepared as live wet mounts and observed using an Olympus BX-51 upright research microscope configured for bright field and DIC microscopy with plain fluorite objectives at 400× to 1000× total magnification. For egg measurements, the length and width was recorded for 30 eggs. For larvae, the length and width of the preseptum, postseptum, pseudointestine and stylets was measured for 30 larvae following the protocols of Szmygiel et al. (2014). Measurements of egg and larval characteristics were taken by capturing digital images of eggs and larvae using an Olympus 5 megapixel digital camera and ImageJ software to obtain measurements (Schneider et al. 2012). In addition, the morphology of the psuedointestine was recorded for larvae.

**Larval preparation for SEM and external larval characteristics.** Poly-L-Lysine coated cover-slips were placed in 1.5 ml plastic well plates. Frozen and live larvae were then thawed, suspended in water, then pipetted onto the Poly-L-Lysine coated cover-slips and fixed in 10 % neutral buffered formalin. Poly-L-Lysine coated cover-slips with fixed larvae were dehydrated in a graded series of ethanol by first placing the Poly-L-Lysine coated cover-slip with fixed larvae in a 1.5 ml plastic well with 0.5 ml of 30 %, 50 % and 70 % ethanol for 30 min each. Next, 1 ml of 100 % ethanol was dripped into the well over a period of an hour, 1 ml of ethanol was then removed from the well and the process repeated 3 additional times. Finally, specimens were dried using HDMS, mounted on aluminum stubs, coated with gold palladium, and examined with a FEI Quanta 600 field emission gun ESEM (ThermoFisher Scientific, Hillsboro, OR) with Evex EDS and HKL EBSD as described previously (Szmygiel et al. 2014). The following morphological surface characteristics were recorded for at least 30 individual larvae: number of terminal spines on the postseptum, the number and relative size of cuticular hooks on the preseptum, the proboscis orientation (dorso-ventrally or laterally compressed) and the number and orientation of spines on the proboscis. Morphological characteristics for larvae examined with SEM followed terminology by Szmygiel et al. (2014).

**Infection of snails to obtain cysts.** Hatched larvae were collected with a Pasteur pipette and approximately 100 larvae were pipetted into 48 1.5 ml well plates filled with 1 mm of aged tap water. Four species of laboratory reared snails from three families maintained at Oklahoma State University following the protocol of Gustafson et al. (2015) were used for infections. Snail species included Physa (Physella) gyrina (Say, 1821), *Stagnicola elodes* (Say, 1821), the M line of *Biomphalaria glabrata* (Say, 1818), and *Planorbella trivolvis* (Say, 1817). For each snail species, 10 individuals were used for exposures, and a single laboratory reared snail was added to each well. Snails fed on the larva mixture for 48 hrs, were then removed and maintained based on species in 3.75 L jars filled with aerated aged tap water with a calcium gravel substrate. Snails were fed on a diet of frozen lettuce and Tetra Min® fish food and gordiid cysts were allowed to develop over a period of four weeks post exposure. Every seven days post exposure (DPE) a few individuals of each snail species were placed in labeled and capped 50 ml plastic centrifuged tubes, filled with approximately 35 ml of aged tap water, and frozen at -80 °C following the protocol of Bolek et al. (2013). Before dissection, centrifuge tubes were thawed and all snails were removed. Snails were processed for gordiid cysts following Harkins et al. (2016). Briefly, the snail body was removed with forceps from its shell under a dissection microscope and then pressed between two slides. Once snail tissue was flattened, a wet mount was prepared by removing the top slide and adding a drop of water and covering the flattened tissue with a cover-slip. Slides were then examined with an Olympus BX-51 upright research microscope (Olympus, Tokyo, Japan) configured for bright field and differential interference contrast microscopy with plain fluorite objectives and a calibrated ocular micrometer at 100× to 400× total magnification. The status and degree of infection were determined by scanning the entire flattened snail carcass for cysts at 100× to 400× total magnification. Twenty cysts were digitally photographed at 1000× total magnification and the length and width of the cyst, cyst wall and encysted larvae were obtained using ImageJ software (Schneider et al. 2012) as previously described for larvae. Morphological characteristics for cysts followed terminology by Harkins et al. (2016).

**Infection of crickets to obtain adults.** Since *P. varius* and *P. obamai* can be domesticated by use of *Acheta domesticus* crickets as definitive hosts (Hanelt et al. 2012; Hanelt and Janovy 2004b), we experimentally-exposed *A. domesticus* to *P. amicus* sp. n. cysts from *Physa acuta* snails. Methods as outlined in Hanelt et al. 2012 were followed.

### Molecular methods

A 1.0 cm mid-section piece, approximately 0.5–2.0 g, was cut into small pieces, dried at room temperature, and DNA was extracted using the E.Z.N.A.^®^ Mollusc DNA Kit (Omega Bio-Tek, Norcross, Georgia), following manufacturer instructions. DNA yield was determined using a NANO DROP 2000c spectrophotometer (Thermo Scientific, Walthem, MA). The *Paragordius* specific cytochrome c oxidase I (CO1) gene was amplified using modified universal CO1 primers (Folmer et al. 1994) Paragordius_cox1F: GGT TAT AGA AAT ACA CAC TCC ATC TT and Paragordius_cox1R2: TAA ACT TCA GGA TGA CCC AAA AAA CC. Subsequent PCR reactions used GoTaq Flexi DNA Polymerase (Promega Corp., Madison, Wisconsin) and were done following manufacturer’s instructions. Agarose gel electrophoresis was done using 1.0 % agarose gels, stained with 0.5 % GelRed Nucleic Acid Gel Stain (Biotium, Hayward, California), and visualized for bands on a UV transilluminator. Amplicons were purified by ethanol precipitation and sequenced using the BigDye version 3.1 kit (Applied Biosystems, Foster City, California) on an ABI 3130× sequence analyzer (Applied Biosystems). Both strands of the sequenced DNA fragments were assembled and edited in Sequencer version 5.0 (Gene Codes, Ann Arbor, Michigan).

### Molecular analyses

Partial CO1 sequences were aligned by eye; no sequences contained indels. As outgroups, two previously-published sequences from *Paragordius* spp. (Table 1) were included, as well as *P. varius* samples from across the United States. Evolutionary history was inferred by using the maximum likelihood method based on the Kimura 2-parameter (K2P) model (Kimura 1980) in MEGA7 (Kumar et al. 2016). All positions containing missing data were eliminated leaving a total of 437 positions in the final dataset. Internal support was assessed using 1,000 bootstrap replicates. CO1 genetic distances between each pair of samples were calculated using the K2P model in MEGA7. This dataset included 658 base pairs. Data were summarized for within and between genetic groups.

## Results

### Taxonomy

#### Paragordius amicus sp. n.

Taxon classificationAnimaliaGordioideaChordodidae

http://zoobank.org/6C823753-BBBB-4749-B29F-31FFCF4ED784

##### Type locality.

Huachuca Mountains, Sunnyside, Arizona, USA (31.445, -110.402, elevation: 1770 m).

##### Holotype.

Female collected on 27 July, 2011, from type locality (N291A). Deposited into the Museum of Southwestern Biology (MSB) Parasite Division, University of New Mexico (UNM), New Mexico, USA with accession number MSB:Para:26387.

##### Paratypes.

Allotype: male specimen collected on 27 July, 2011, from the type locality (N291B). Deposited into the MSB Parasite Division, accession number MSB:Para:26388. Paratypes: two females collected 28 July 2011, in the Santa Rita Mountains (N289A, and N289B). Deposited into the MSB Parasite Division, accession numbers MSB:Para:26389 and MSB:Para:26390.

##### Host.

Natural definitive insect host is unknown; in the laboratory, *Acheta domesticus*, crickets served as definitive hosts, but in nature are likely to be members within the family Gryllidae (crickets) or Tettigoniidae (bush-crickets or katydids).

##### Etymology.

The name is Latin for “friend”, referring to the fact this is the first description of another genus member for *P. varius* in the Nearctic.

##### Distribution.

Current known distribution is limited to the Madrean Sky Islands of southeastern Arizona in the Huachuca and Santa Rita Mountain Ranges.

##### Material examined.

Adults (n=5), eggs, larvae, and cysts. Tissue from field collected adult (N=4) midsections was utilized for CO1 analysis while adult posterior, anterior, and midsections were utilized for SEM. DNA was also extracted from a worm removed from a deceased, lab-infected *Acheta domesticus* 40 days post exposure to collected larvae. Egg, larvae, and cyst stages were imaged using SEM and/or DIC microscopy.

##### Description of male.

**Figure 1.**
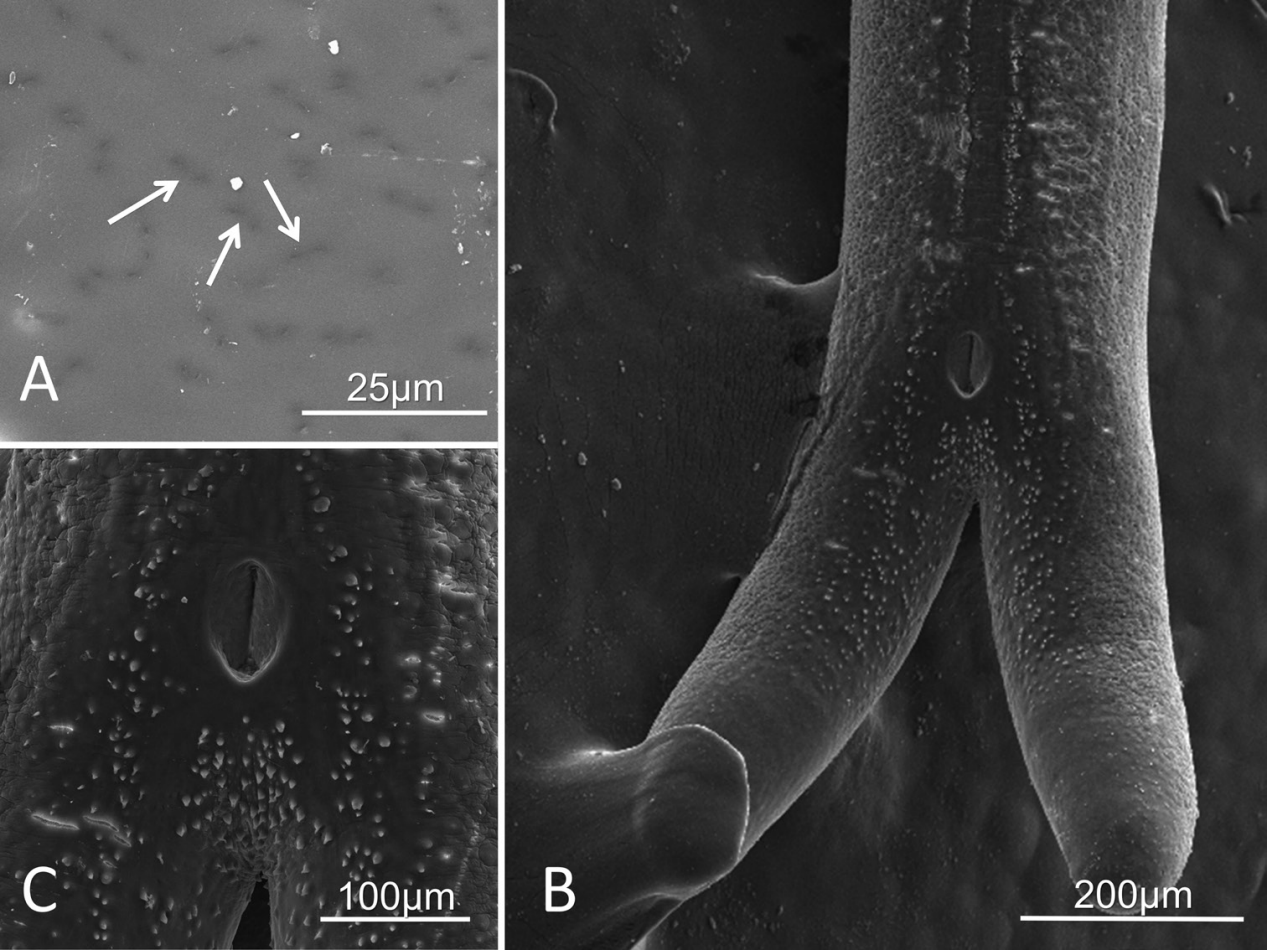
*Paragordius amicus* sp. n. adult male from the Huachuca Mountains. **A** Midbody cuticle showing lack of obvious surface structure; some superficial structure is seen (arrows) **B** Bifurcating posterior end exhibiting the characteristic male bifurcating lobes with small, circular pointed lobes extending from the base of the bifurcation up the interior side of the lobes. These mounds are also found on either side as well as below the cloacal opening, eventually merging into the border of the ventral smooth cuticle line. **C** Cloacal opening, oblong with a straight slit opening extending the length of the opening surrounded by spines.

Adult (n=1) 205 mm long medium brown color. Bifurcating tail lobes on posterior roughly 400 µm in length, extending laterally away from the sagittal plane (Fig. 1B). Male cloacal opening oval with a vertical slit-like opening (30×60 µm, located anterior to point of tail lobe bifurcation (Fig. 1C). Post-cloacal spines present just above bifurcation but posterior to the cloaca extending onto the inside and ventral side of the tail lobes (Fig. 1B, C). Midbody cuticle lacks of obvious surface structures; some superficial structure is noted as dark-appearing areas (Fig. 1C). Cuticle on posterior end has wrinkled appearance made of grooves and circular pattern and is evenly dispersed across the cuticle surface with the exception of a 25 µm wide line running on the ventral surface, along the sagittal line, lacking grooves or circular patterning. Smooth ventral line of cuticle is bordered by small pointed mounds approximately the same size and shape as postcloacal spines but are mound rounded and mound-like (Fig. 1A, C).

##### Description of female.

**Figure 2.**
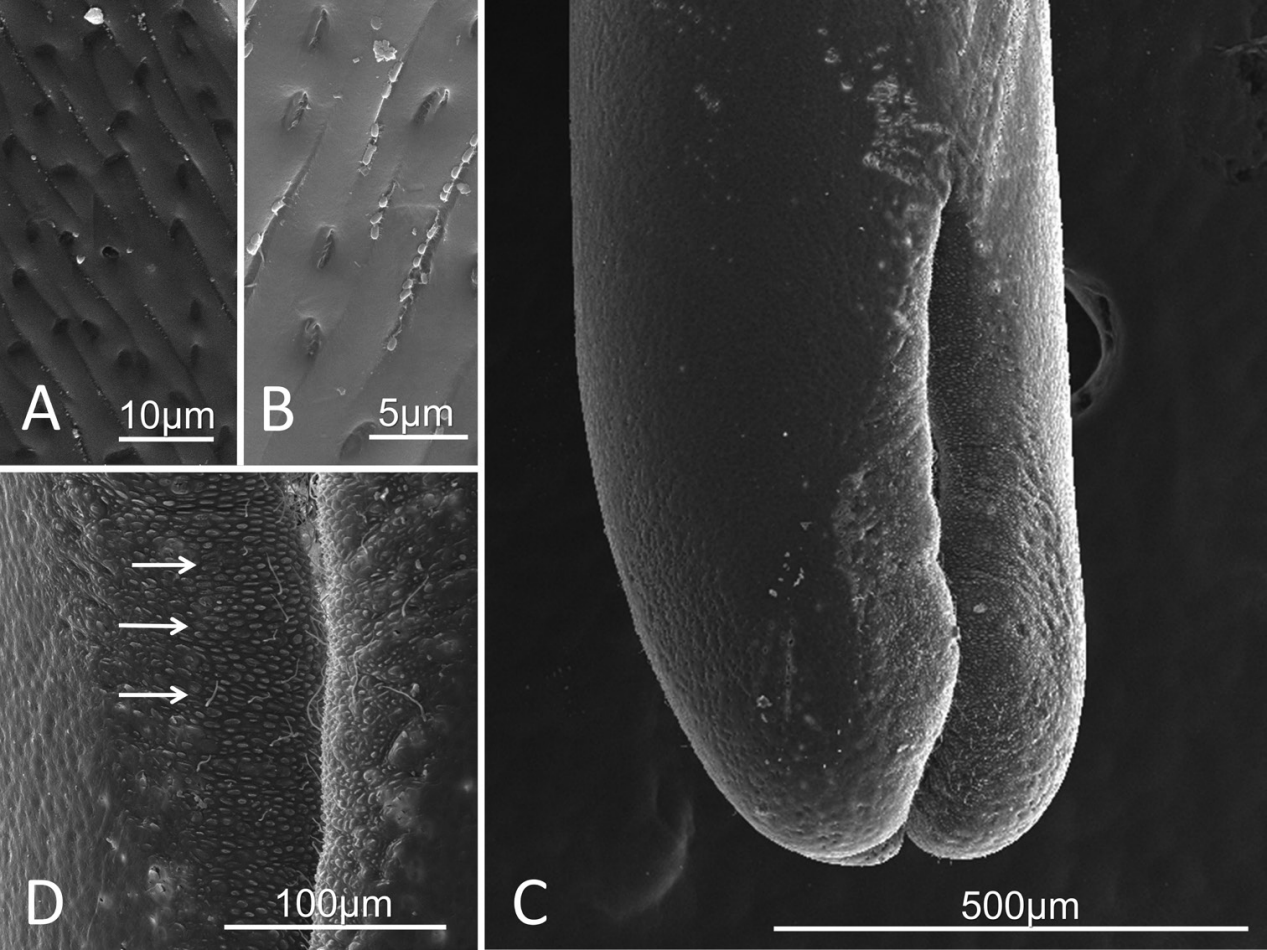
*Paragordius amicus* sp. n. adult female from the Santa Rita Mountains **A–B** Midbody cuticle exhibiting transverse striations made of ridges separated by furrows **A** In some areas with hollow round to oblong indentations **B** In other areas with more oblong indentations containing structures **C** Trifurcating posterior. Note the cuticle features that can be seen on the interior surface of the lobes **D** Increased magnification of the lobes showing the oblong mounds (arrows). Note the long and thin hairs extending from some of the furrows between the mounds.

**Figure 3.**
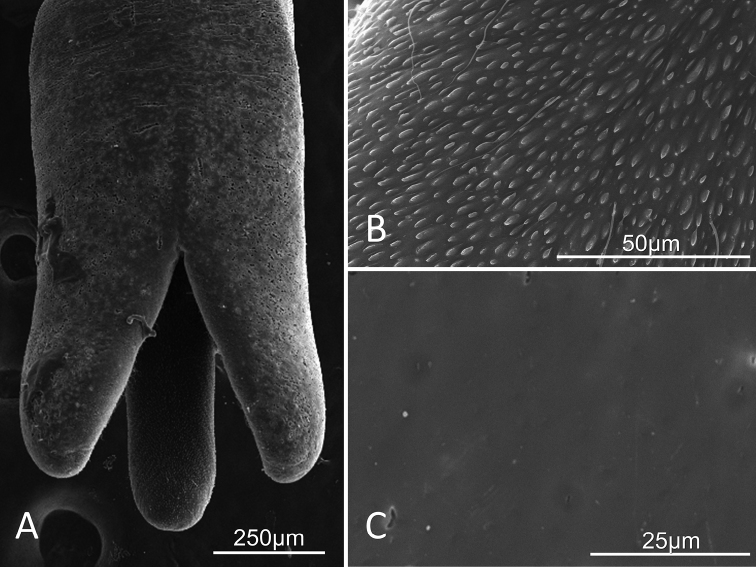
*Paragordius amicus* sp. n. adult female from the Huachuca Mountains **A** Trifurcating posterior. **B** High magnification view of the oblong mounds and long, thin hair extensions found on the interior surface of the trifurcating lobes **C** Midbody cuticle lacking obvious surface structure.

**Figure 4.**
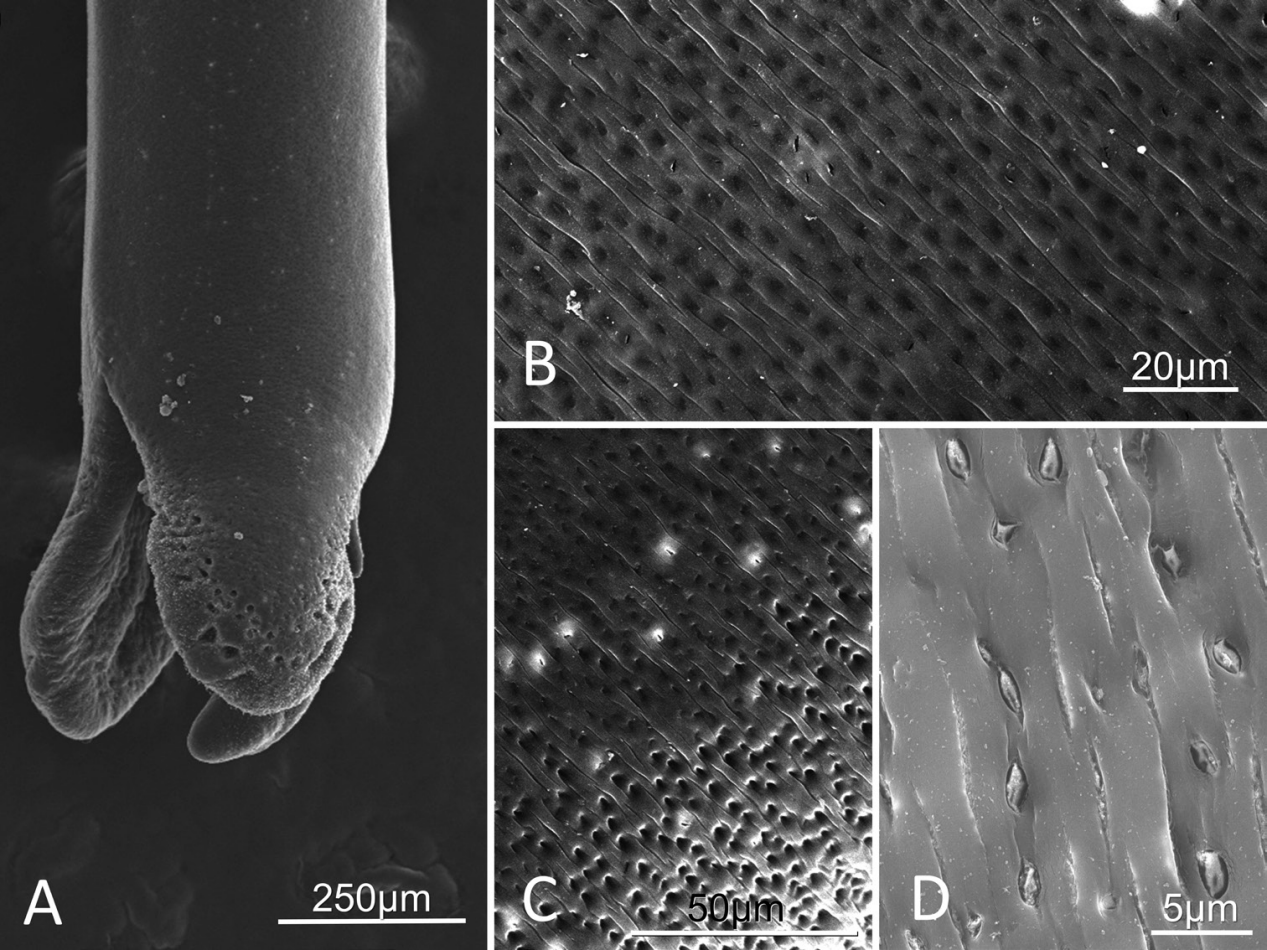
*Paragordius amicus* sp. n. adult female from the Santa Rita Mountains **A** Trifurcating posterior **B–D** Midbody cuticle surface of with transverse striations containing round to slightly oblong indentations **B–C** Areas with only hollow indentations **D** Area of cuticle with more oblong indentations housing structures.

Adults (n=3) were 198 mm, 216 mm, and 234 mm in length and medium brown in color. Trifurcated posterior end (Figs 2C, 3A, 4A), with varying degrees of openness. Distinctive oblong mounds, approximately 10µm in length, are found arranged in horizontal or vertical lines up and down the entire interior side of the trifurcating tail lobes (Figs 2C, D, 3A, B). Short, thick bristles (hair-like structures) randomly spaced between oval mounds (Figs 2D, 3B). Midbody cuticle geographically variable. Worms collected in the Santa Rita Mountains contained transverse striations consisting of raised ridges separated by narrow furrows (Figs 2A, B, 4B, C, D). In some areas, the cuticle also contains rounded indentations (Figs 2A, 4B, C), while in others the indentations were more oblong and housed structures (Figs 2B, 4D). The female collected from the Huachuca Mountains, just as the male collected from the Huachuca Mountains, lacked any obvious surface structure on the midbody cuticle (Figs 1A, 3C).

##### Description of egg strings, eggs, and larvae.

**Figure 5.**
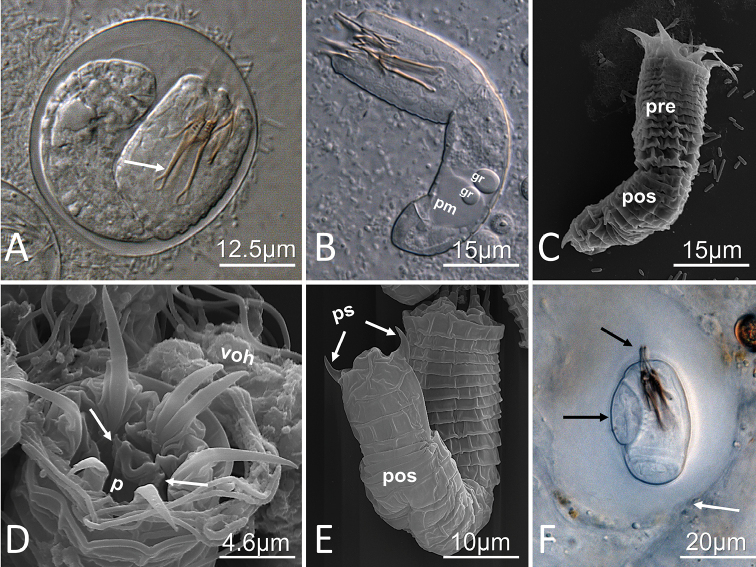
Egg, larva and cyst characteristics of *Paragordius amicus* sp. n. **A** DIC photomicrograph of an egg with a fully developed larva. Note the stylets (arrow) **B** DIC photomicrograph of a live larva. Note the pseudointestine composed of two anterior granules (gr) and posterior mass (pm) **C** SEM photomicrograph of larva. Note the distinct preseptum (pre) with relatively long outer hooks, and postseptum (pos) with posterior spines **D** SEM photomicrograph of the anterior end larva. Note the partially everted proboscis (p) with distinct spines on the left and right lateral sides (white arrows), and the outer row of hooks containing two ventral outer hooks (voh) **E** SEM photomicrograph of larva. Note two posterior spines (ps) on the dorsal side of the postseptum (pos) **F** DIC photomicrograph of cyst (dorso-ventral view). Note the clear cyst wall (white arrow), distinct spines on the preseptum (black arrows), and the position of the posterior end of the postseptum (white arrow).

Females deposited continuous egg strings that were white in color and 1–3 times the length of the females. Eggs were elliptical to spherical in shape with a thin shell and were 36.6 (29.6–41.2) µm in length and 32.0 (25.9–43.8) µm in width. Over a period of 3–4 weeks, egg strings turned a light brown color at which time eggs contained fully developed larvae (Fig. 5A).

Larvae possessed a cylindrical body divided by a septum into two regions, the preseptum and a postseptum (Fig. 5B, C). The preseptum was 29.4 (24–38) µm in length and 15.4 (13–17) µm in width and contained an eversible proboscis supported by three internal stylets which were 13.8 (12– 16) µm in length and 4.5 (3–6) µm in width (Fig. 5A, B). The postseptum was 34.8 (29–39) µm in length and 12.6 (10–15) µm in width and contained a clearly visible pseudointestine. The pseudointestine contained two anterior granules and a large posterior mass and was 15.5 (11–18) µm in length and 10.1 (7–13) µm in width (Fig. 5B). The average preseptum to post septum ratio was 1:1.2 (1.1–1.5).

Externally, larvae were superficially annulated and the postseptum contained two pairs of terminal spines located ventrally (Fig. 5C, E). The preseptum contained three sets of cuticular hooks. The outer ring of hooks contained seven hooks, two of which are very close together and ventrally positioned (Fig. 5D), and there were six hooks in the middle and inner rings observed in live larvae. The length of the cuticular hooks on the outer ring was noticeably longer than the middle and inner cuticular hooks. Clearly visible spines on the proboscis could only be observed in a few individuals (Fig. 5D). The left and right side of the distal end of the dorsoventrally compressed and eversible proboscis each contained spines (at least four pairs arranged in tandem and one single spine above); whereas the distal end of the ventral side of the proboscis contained five spines (two pairs arranged in tandem and one single spine above; Fig. 5D).

**Table 1.** Collecting location for hairworm samples used in this study.

Species/sample	Accession^†^	Collection location^‡^	Lat.	Long.	Genbank accession
*Paragordius amicus* sp. n.					
N289A	MSB:Para:26389	Arizona	31.713	-110.874	MG654049
N289B	MSB:Para:26390	Arizona	31.713	-110.874	MG654050
N291A	MSB:Para:26387	Arizona	31.445	-110.402	MG654047
N291B	MSB:Para:26388	Arizona	31.445	-110.402	MG654048
*Paragordius varius*					
N000	MSB:Para:26391	Nebraska	40.994	-96.566	MG654052
N138^§^	MSB:Para:26392	Montana			MG654053
N210	MSB:Para:26393	Missouri	37.300	-89.550	MG654054
N256	MSB:Para:26394	Mississppi	34.359	-88.462	MG654055
N364A	MSB:Para:26395	New Mexico	34.766	-106.328	MG654056
N364B	MSB:Para:26396	New Mexico	34.766	-106.328	MG654057
N398		Oklahoma			KU721073
Outgroups					
*Paragordius* sp.^§^		South Africa			AY428843
*Paragordius obamai*	MSB:Para:26397	Kenya	-0.152	34.446	MG654059
*Paragordius tricuspidatus*	MSB:Para:26398	France	43.755	3.110	MG654058

^†^ Museum of Southwestern Biology, Parasitology Division. ^‡^ Within the United States unless otherwise noted. ^§^ Exact locality is unknown.

##### Laboratory rearing of cysts and adults, and description of cyst.

Of the four snail species exposed to larvae of *P. amicus* sp. n. only *Physa acuta* and *Biomphalaria glabrata* became infected with cysts; however, not all individuals became infected. Seven of 10 (70 %) *P. acuta* were infected with a mean abundance of 2.8 ± 3.0 (range 0–8) cysts; and four of 10 (40 %) of *B. glabrata* were infected with a mean abundance of 1.0 ± 2.2 (0–7) cysts.

Fully developed cysts were recovered from laboratory-reared and exposed snails 14–21 DPE. They contained a clear cyst wall of unknown composition 16.1 (12–24) µm in length and 11.5 (9–13) µm in width (Fig. 5F). During cyst formation the content of the larval pseudointestine was emptied and the larva folded its postseptum twice around the preseptum. However, unlike cysts of other gordiid genera, the posterior end of the postseptum never reached the posterior end of the preseptum (Fig. 5F). The folded larva inside of the cyst was 28.1 (26–29) µm in length and 18.6 (18–20) µm in width.

Of approximately 40 *A. domesticus* crickets exposed to about 10–100 cysts each, approximately 7 worms developed in 4 cricket hosts. To establish that the parasite infection was *P. amicus* sp. n. one worm was extracted for DNA, amplified, and sequenced as described above. The sequence was 100 % identical to both worms sequenced from the Santa Rita Mountains, and was placed into Genbank as MG654051.

##### Diagnosis and taxonomic comments.

*Paragordius amicus* sp. n. has unique morphological features which warrant placing it as a new species and make it distinct from other New World *Paragordius*. First, the semi-oval raised cuticle structures and the short bristles (hair-like structures) on the inside of the female tail lobes have not been documented previously in *Paragordius* species. Second, despite the geographical variation in the cuticle structure of *P. amicus* sp. n., both variants have a cuticle pattern not seen in Nearctic and New World *Paragordius* species. *Paragordius varius* is the only species in the Americas also containing transverse striations separated by furrows. However, in *P. varius* the ridges within the striations are topped with round knobs (Schmidt-Rhaesa et al. 2003). *Paragordius flavescens* Linstow, 1906, found in South America, and *P. diversolobatus* Heinze, 1935 from Costa Rica contain areoles. *Paragordius esavianus* Carvalho, 1942, from South America lacks areoles but the cuticle is covered by dispersed round tubercles, longer bristles, and irregular small cuticular elevations. *Paragordius minusculus* Carvalho, 1944, found in Brazil, lacks areoles but the midbody cuticle is fully covered by digit-like cuticular projections, like bristles, with blunt apexes. Finally, *Paragordius andreasii*, Zanca & de Villalobos, 2006, from Argentina, has a midbody cuticle with oval or rounded depressions arranged in pairs or forming perpendicular lines to the axis of the body. Finally, the female *P. obamai* Hanelt et al., 2012, does contain structures on the inside of the tail lobes. However, these structures are longitudinal, parallel ridges from which more narrow and longer bristles emerge (Hanelt et al. 2012).

Morphological characteristics of egg stings, eggs, larvae, and cysts of *Paragordius amicus* sp. n. were indistinguishable from these non-adult stages of two other species of *Paragordius* (*P. obamai* and *P. varius*) for which non-adult descriptions exist (Szmygiel et al. 2014). However, egg strings, larvae, and cysts of *Paragordius amicus* sp. n. were morphologically distinct from egg strings, larvae of other gordiid genera such as *Acutogordius*, *Chordodes*, *Gordius* and *Neochordodes* (Chiu et al. 2017; Szmygiel et al. 2014).

##### Molecular data.

**Figure 6:**
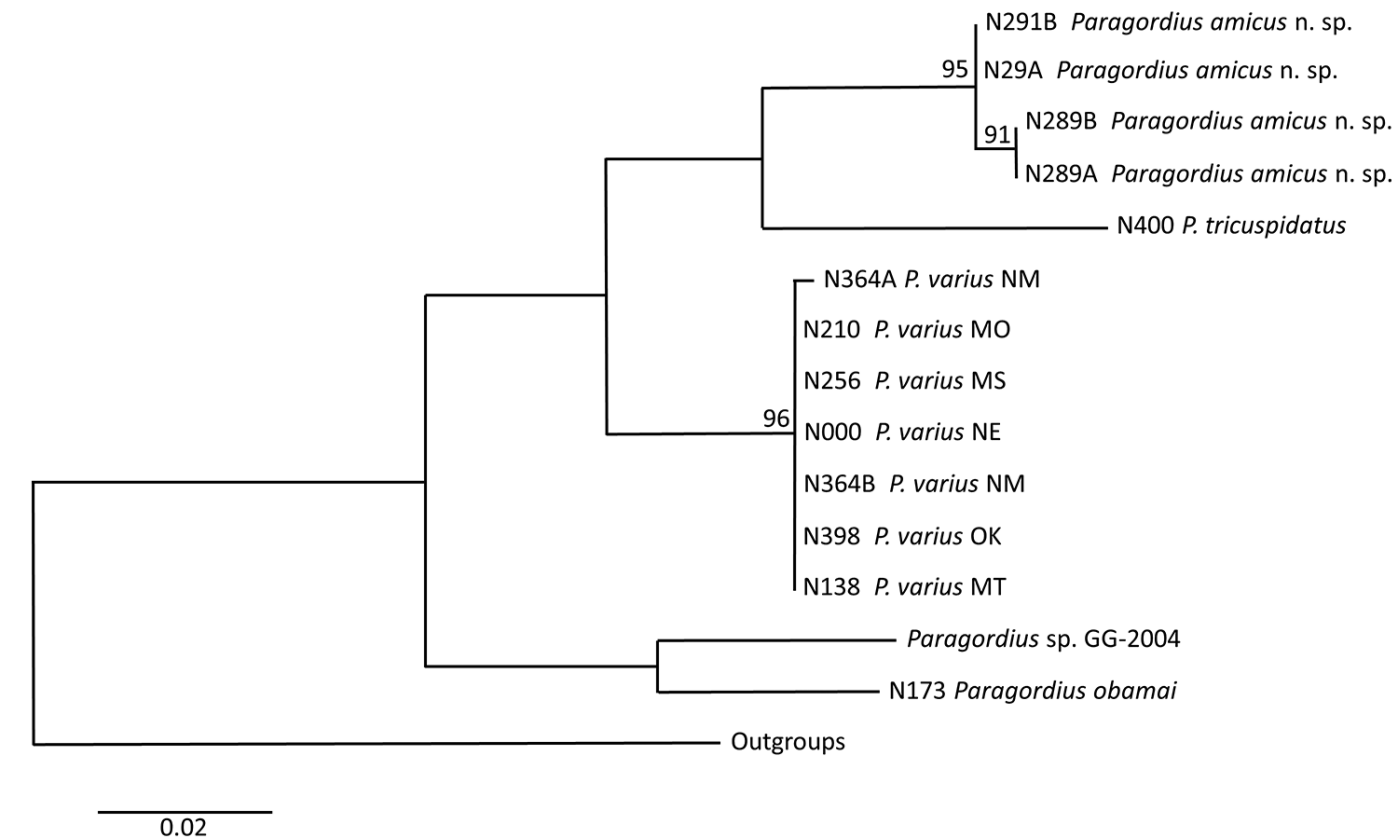
. Relationships inferred by maximum likelihood. Tree is unrooted, and drawn to scale indicating number of substitutions per site. Bootstrap values above 0.90 are shown.

Genetic distances of the CO1 barcoding region supports our contention that *P. amicus* sp. n. is a new species and that it is distinct from *P. varius*. The intraspecific distances among *P. varius* samples from around the USA is 0.72 %, while among *P. amicus* sp. n. samples is 1.09 %. The interspecific distance between *P. varius* and *P. amicus* sp. n. is 25.33 %. The inferred phylogenetic relationship (Fig. 6) supports the clustering of *P. varius* from around the USA forming a monophyletic group.

## Discussion

*Paragordius amicus* sp. n. represents the first hairworm described as an endemic to the Madrean Sky Islands, and so far only the second new species to be documented form the Chiricahua Mountains (see also Hanelt et al. 2015). There has been increased need for diversity studies like this in light of the emerging global crisis of climate change, especially for parasites which are chronically understudied organisms (Carlson et al. 2017; Dougherty et al. 2016). Climate change is considered the greatest threat to biodiversity, and its affects are often disproportionate depending upon an organism’s life history, distribution, and location (Dunn et al. 2009; Malcolm et al. 2006). Due to their dependence on aquatic and terrestrial habitat and the spatial and temporal synchronization of paratenic and definitive hosts, hairworms may be a group heavily impacted by the effects of climate change.

The Madrean Sky Islands are considered a biodiversity hotspot, or “cradles of diversity”, and thus climate change may have a proportionally large impact on this ecosystem (Malcolm et al. 2006; McCormack et al. 2009; O’Neal et al. 2005). The Sky Islands are actually expected to experience the influences associated with climate change sooner than other parts of the west (Garfin et al. 2013; Notaro et al. 2012). The most immediate of these impacts is wildfire. In the Western U.S.A, the average annual area affected by wildfires has increased by more than six fold over the past four decades (Littell et al. 2009; Westerling et al. 2006). Indeed, since collecting the specimens described in this study, several fires have swept both the Santa Rita Mountains (e.g. Sawmill fire, April 2017, 47,000 acres) and the Huachuca Mountains (e.g. Monument fire, June 2011, 29,000 acres).

Like oceanic islands, Sky Islands are habitat surrounded by barriers to biological dispersal. These barriers lead to isolation and ultimately high rates of endemism. Although *Paragordius amicus* sp. n. is not isolated to a single island, we have tantalizing evidence that the populations on the Santa Rita Mountains and Huachuca Mountains, separated by just 53.5 kilometers, may have been temporally isolated. These two populations appear to vary morphologically, in their cuticle pattern, but also are separated genetically by about 1.1%. Comparatively, *P. varius*, collected from several locations separated by hundreds and up to 1,600 kilometers apart vary genetically by only an average of 0.72%. In the future, we hope to collect additional specimens to more thoroughly document geographical variation in morphology and genetics.

## Supplementary Material

XML Treatment for Paragordius amicus
